# Idiopathic Thrombosis of the Inferior Vena Cava and Bilateral Femoral Veins in an Otherwise Healthy Male Soldier

**DOI:** 10.1155/2013/246201

**Published:** 2013-09-25

**Authors:** Sarah Gordon, Tamie Kerns, William Londeree, Brian Ching

**Affiliations:** ^1^Department of Medicine, Tripler Army Medical Center, 1 Jarrett White Road, Honolulu, HI 96859, USA; ^2^Hematology-Oncology Service, Tripler Army Medical Center, 1 Jarrett White Road, Honolulu, HI 96859, USA; ^3^Department of Radiology, Tripler Army Medical Center, 1 Jarrett White Road, Honolulu, HI 96859, USA

## Abstract

Thrombosis of the inferior vena cava is less common than deep venous thrombosis of the lower extremities, particularly in the absence of an obvious congenital caval abnormality or hypercoagulable state. We present a case of IVC thrombosis in an otherwise healthy and active 28-year-old male soldier secondary to dehydration and venous webbing. IVC thrombosis is an uncommon and underrecognized condition; in this case, the patient's caval thrombosis was initially mistaken for acute back strain. Prompt recognition is necessary to minimize long-term sequelae.

## 1. Introduction

Thrombosis of the inferior vena cava associated with filter placement is a well-described entity, but thrombosis of the inferior vena cava (IVC) is rare in otherwise healthy adults [1]. Virchow's triad of hypercoagulability, endothelial injury, and venous stasis applies as it does to peripheral deep venous thrombosis. Hypercoagulability related to hematological or neoplastic processes, venous stasis secondary to compression from a tumor, hematoma, or infectious process, and endothelial injury due to trauma or foreign body have all been implicated in the pathophysiology of IVC thrombosis. In the older patient population, intra-abdominal malignancy and presence of an IVC filter are prevalent underlying causes. In younger patient populations, a heritable hematologic abnormality or congenital abnormality should be strongly considered [2]. 

Because thrombosis of the IVC is uncommon, it may not be recognized until the affected patient develops severe symptoms. It is associated with a higher risk of complication than other forms of deep venous thrombosis [3]. In the acute setting, pulmonary embolism is a concern, as is renal and hepatic vein thrombosis. Long-term patients can suffer from recurrent lower extremity venous thrombosis, continued edema, and postphlebitic syndrome. Prompt diagnosis and treatment of this disorder are necessary to prevent complications.

## 2. Case Report 

A twenty-eight-year-old Caucasian male soldier presented to primary care with the complaint of lower back pain for two-day duration. His pain began three days after running a ten mile race with his military unit. During the race, he reported feeling dehydrated but denied any falls or injuries. He had no past medical history, and his only previous surgery was a right inguinal hernia repair performed at age twenty-five. The patient denied recent trauma, surgery, immobilization, or family history of thrombotic disorder. His only medication was ibuprofen as needed. He was an active smoker of one pack per week for the past three years and reported only social use of alcohol. 

The patient's vital signs were within normal limits, and his exam was notable only for lumbar paraspinal muscle tenderness. A lumbar spine plain film showed no abnormalities. He was diagnosed with musculoskeletal lumbar pain and provided with a nonsteroidal anti-inflammatory drugs. Ten days after the onset of pain, he noticed lower extremity swelling and returned to care. At this time, he was noted to have moderate to severe pitting edema in the bilateral lower extremities to the mid-thigh, worse on the right, and dependent erythema bilaterally. 

A deep venous thrombosis was suspected, and the patient was referred to the emergency department for expedited workup. A fibrin d-dimer was elevated at 7.74 mcg/mL. Blood counts were notable for a normocytic anemia with hemoglobin 10.9 g/dL, hematocrit 32%, and mild thrombocytopenia (platelet count 100,000/*μ*L). A urinalysis showed 1+ hematuria and no proteinuria. Additional studies including hepatic function panel, electrolyte panel, and brain natriuretic peptide were within normal limits. Doppler ultrasounds of the lower extremities were performed with no evidence of venous thromboembolism visualized. A transthoracic echocardiogram and electrocardiogram did not demonstrate any abnormalities. 

**Figure 1:**
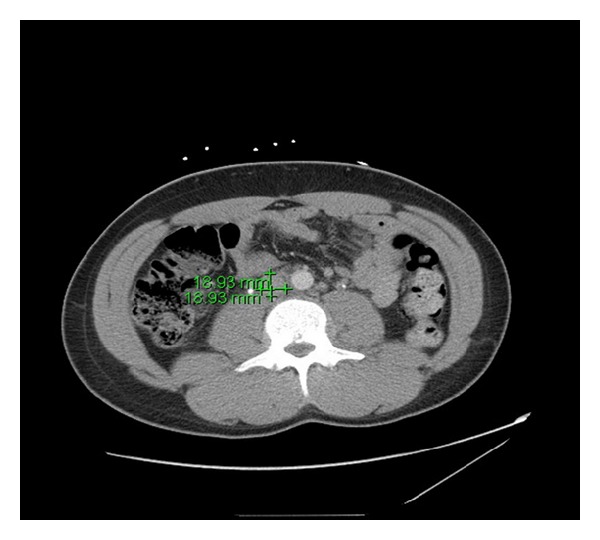
Dilated abdominal veins and IVC thrombosis visible on computed tomography.

A contrast CT of the abdomen and pelvis demonstrated diffusely dilated veins of the lower abdomen and pelvis, suggestive of a proximal venous obstructive process. A triphase liver CT ruled out hepatic congestion and Budd-Chiari syndrome but showed a long segment filling defect involving the infrarenal IVC (Figure 1). MRI abdomen was performed to characterize the extent of clot burden and clarify anatomy. It confirmed thrombus in the right common femoral vein, left distal external iliac vein into the infrarenal inferior vena cava. There was no evidence of involvement of the renal veins. 

Because of the patient's young age, the large size of his thrombosis, and the severity of his symptoms, localized thrombolysis with tissue plasminogen activator was attempted. Follow-up venogram showed only minimal improvement in venous flow. The patient was anticoagulated with warfarin and was referred for caval and bilateral iliac drug eluting stents. After the procedure, good return of flow was noted; the patient had decreased swelling and pain, and his mild anemia and thrombocytopenia were corrected. He was subsequently anticoagulated with a planned 3 months of antiplatelet treatment with clopidogrel and 3–6 months of anticoagulation with warfarin. He did continue to have some lower extremity pain, and he was treated with oxycodone-acetaminophen, venlafaxine, and gabapentin. He ultimately required a referral to the pain management service. He continued to have persistent lower extremity pain and edema, though his symptoms are less than at initial presentation. 

Four months after initial stenting, he developed unilateral recurrence of his symptoms and rectal bleeding. He was treated with restenting. His hemoglobin and hematocrit remained at baseline, and his rectal bleeding was resolved spontaneously without cessation of anticoagulation. His warfarin duration is now extended to lifelong anticoagulation. 

Because of the extensive nature and unusual location of the patient's thrombosis, an evaluation was performed for hematologic disorders. Laboratory assessment of protein C and S activity, cardiolipin antibodies, factor V Leiden activity, direct Russell's viper venom test, antithrombin III activity, prothrombin gene mutation, methylenetetrahydrofolate reductase gene mutation, and b2 glycoprotein was unrevealing. Malignancy was ruled out with physical exam and imaging. 

## 3. Discussion

Idiopathic IVC thrombosis is unusual. The lifetime incidence of all venous thrombosis is estimated at 0.1%; 20% of affected patients have no clear precipitating factor identified [2]. IVC thrombosis is less common than deep venous thrombosis of the lower extremities and is rarely idiopathic [4]. It can result from extension of iliofemoral or pelvic vein thrombosis, by primary thrombosis in the setting of a hematologic abnormality, and through mechanical obstruction by a caval filter or malignancy [5]. This patient had no hematologic abnormality identified but had a severe thrombosis and was treated unconventionally with thrombolysis and stenting in addition to anticoagulation. 

We considered a variety of causes in this patient, including a heritable hematologic abnormality, paroxysmal nocturnal hemoglobinuria (PNH), which can present with proximal thrombosis in up to 12% of affected patients, extension from an iliofemoral thrombosis, and May-Thurner syndrome, which presents with recurrent deep venous thrombosis [5, 6]. He had a negative workup for hematologic abnormalities; however, labs were drawn at the time of a large clot burden. The fact that his initial symptom was back pain and his bilateral iliofemoral thrombosis suggests that in this case IVC thrombosis preceded the iliofemoral component. His hematuria was proved to be transient and testing for PNH was negative.

Caval abnormalities are another well-described risk factor for thrombosis. The normal IVC is composed of four segments which are formed from anastomoses of three pairs of embryonic veins. The complexity of this embryologic process leaves ample opportunity for abnormalities in regression or persistence of the embryonic veins, which can then promote thrombosis later in life. Prevalence of these congenital abnormalities ranges from 0.2 to 3% in the general population. In patients with any form of deep venous thrombosis, the rate is higher, 5–16% [7]. It is unclear if a caval malformation alone can precipitate IVC thrombosis; some authors report this to be true; however, in most cases, a combination of anatomic and exogenous factors contributes [7–11]. Lifelong anticoagulation is often required because in the absence of a reversible precipitating cause, presumptive congenital caval abnormality is associated with continued risk of IVC thrombosis. This patient had evidence of venous webbing and stenosis during venography, but no evidence of IVC segment absence. The etiology of his thrombosis appears to be congenital atresia of the IVC with inadequate collateral drainage in the pelvic region. He likely had a concurrent hypercoagulable state induced by dehydration. 

 Regardless of the cause, prompt recognition of IVC thrombosis is important because of the potential acute complications. It carries a higher risk of pulmonary embolism than lower extremity deep venous thrombosis with 33% reported [3]. There is also risk associated with clot propagation including extension to the renal veins and extension to the hepatic veins. Though rarely reported, critical limb ischemia secondary to phlegmasia cerulea dolens is another potential complication. Finally, septic thrombus can also be life-threatening [12]. 

 The Wells score is recognized as a method to risk stratify patients suspected to present with deep venous thrombosis, but no equivalent validated scoring system exists for caval thrombosis [13]. Physical exam findings and symptoms are variable and dependent on the degree and location of occlusion. Once suspected, the diagnosis of IVC thrombosis is established through imaging. Computed tomography (CT) with contrast and magnetic resonance imaging (MRI) has been shown to be equally sensitive [14]. The gold standard imaging modality is venography, though this is invasive and time consuming. It is the preferred method if surgical intervention is planned. In this case, a CT identified and venography confirmed the presence of caval thrombosis.

 Sources agree that immediate anticoagulation improves morbidity and mortality by reducing risk of pulmonary embolism and propagation of clot. Catheter directed thrombolysis has largely replaced surgical thrombectomy, and balloon dilation and endovascular stent placement are also alternatives. Thrombosed venous segments which fail to recanalize rapidly undergo subsequent fibrotic organization which leads to fixed stenosis or occlusion. This process of clot organization can lead to chronic obstructive symptoms or postphlebitic syndrome. Also, 15% of untreated deep venous thromboses will extend proximally [1]. Our patient was treated with thrombolysis followed by anticoagulation and stenting. The accepted indications for this more aggressive treatment include young age, lack of comorbidities, and limb threatening thrombosis. Some reports suggest that valve patency is better maintained after thrombolysis [15]. In this case, thrombolysis and stenting were chosen because of the size of his thrombosis and severity of symptoms, and because the patient wished to remain on active duty status, which chronic anticoagulation would preclude. He did not have any of these potentially deadly complications of acute caval thrombosis. He did however suffer from some of the known late complications, including pain, erythema, and swelling associated with postphlebitic syndrome, and recurrent thrombosis. When stenting and anticoagulation fail, quality of life is often dramatically improved with daily use of compression stockings, a treatment which has been stressed for this patient. 

This patient's case is of interest because IVC thrombosis is rare compared to lower extremity deep venous thrombosis, particularly in young patients and in the absence of an anatomic risk factor or hematologic disorder. It also highlights the importance of early diagnosis since his course was complicated by restenosis and postthrombotic syndrome.
